# Development of a core outcome set for use in determining the overall success of gastroschisis treatment

**DOI:** 10.1186/s13063-016-1453-7

**Published:** 2016-07-27

**Authors:** Benjamin Allin, Andrew Ross, Sean Marven, Nigel J Hall, Marian Knight

**Affiliations:** 1 National Perinatal Epidemiology Unit, University of Oxford, Richard Doll Building, Old Road Campus, Oxford, OX3 7LF England; 2 Oxford University Hospitals NHS Trust, Headley Way, Headington, Oxford, OX3 9DU England; 3 Sheffield Children’s Hospital, Western Bank, Sheffield, S10 2TH England; 4 Faculty of Medicine, University of Southampton, Southampton, SO16 6YD England

**Keywords:** Paediatric surgery, Gastroschisis, Core outcome sets

## Abstract

**Background:**

Gastroschisis research is limited in quality by the presence of significant heterogeneity in outcome measure reporting (PloS One 10(1):e0116908, 2015). Using core outcome sets in research is one proposed method for addressing this problem (Trials 13:103, 2012; Clin Rheumatol 33(9):1313-1322, 2014; Health Serv Res Policy 17(1):1-2, 2012). Ultimately, standardising outcome measure reporting will improve research quality and translate into improvements in patient care.

**Methods/design:**

Candidate outcome measures have been identified through systematic reviews. These outcome measures will form the starting point for an online, three-phase Delphi process that will be carried out in parallel by three panels of experts. Panel 1 is a neonatal panel, panel 2 is a non-neonatal panel and panel 3 is a lay panel.

In round 1, experts will be asked to score the previously identified outcome measures from 1–9 based on how important they think the measures are in determining the overall success of their/their child’s/their patient’s gastroschisis treatment.

In round 2, experts will be presented with the same list of outcome measures and with graphical representations of how their panel scored that outcome in round 1. They will be asked to re-score the outcome measure taking into account how important other members of their panel felt it to be. In round 3, experts will again be asked to re-score each outcome measure, but this time they will receive a graphical representation of the distribution of scores from all three panels which they should take into account when re-scoring.

Following round 3 of the Delphi process, 40 experts will be invited to attend a face-to-face consensus meeting. Participants will be invited in a purposive manner to obtain balance between the different panels. The results of the Delphi process will be discussed, and outcomes re-scored. Outcome measures where > 70 % of the participants at the meeting scored them as 7–9 and < 15 % scored them as 1–3 will form the core outcome set.

**Discussion:**

Development of a core outcome set will help to reduce the heterogeneity of the outcome measure reporting in gastroschisis. This will increase the quality of research taking place and ultimately improve care provided to infants with gastroschisis.

**Electronic supplementary material:**

The online version of this article (doi:10.1186/s13063-016-1453-7) contains supplementary material, which is available to authorized users.

## Background

Gastroschisis is an increasingly common paediatric surgical condition, which now affects approximately 1:3000 live births in the UK [1]. Mortality for infants with gastroschisis is relatively low [2]. However, the initial inpatient stay for these infants is the longest of any of the commonly encountered birth defects [3], and children are often affected by significant long-term morbidity in the form of repeat operations, need for enteral feeding assistance and parenteral nutrition use [2, 4]. The cost to the NHS of managing infants with gastroschisis is also significant, with their initial inpatient stay costing approximately £90,000, and any subsequent unplanned operations under the age of one costing approximately £25,000 [5, 6].

Multiple surgical repair strategies exist for the treatment of gastroschisis, with the two most common being operative primary fascial closure and silo placement with staged reduction and delayed closure [1]. Currently, significant heterogeneity exists in outcome measure reporting in studies comparing these two key interventions for gastroschisis (Allin BSR, Irvine A, Patni N, Knight M: Variability of outcome reporting in Hirschsprung’s Disease and gastroschisis: a systematic review, unpublished). Such heterogeneity has led to studies focusing on surrogate markers of treatment success, short-term outcomes or hospital metrics, as opposed to outcomes that are of relevance to patients or that impact the long-term quality of life [7, 8]. This lack of patient relevance is one of three problems caused by heterogeneity in outcome measure reporting; the other two are a risk of reporting bias and an inability to synthesise data arising from multiple studies.

For gastroschisis, where little research is taking place and where the majority of that research is of a small sample size, reported outcomes should be relevant, accurately represent the studies’ findings and be synthesisable through meta-analysis. If reported outcomes do not meet these standards, the derivation of robust, evidence-based management guidelines for infants with gastroschisis will be impossible. A lack of such guidelines may contribute to the wide variation in management and outcomes for infants with gastroschisis specifically and for infants requiring early surgery more generally [1, 2, 9–11].

The COMET (Core Outcome Measures in Effectiveness Trials) initiative was established to bring together people with an interest in the development and application of agreed upon standardized sets of outcome measures, known as core outcome sets [12, 13]. Core Outcome Sets are groups of outcome measures, commonly identified through a Delphi process and ratified by key stakeholders, as the outcomes that minimally should be reported in every study of a given condition [13]. Core outcome sets have already been developed for conditions as diverse as ankylosing spondylitis and asthma and have been shown to improve the quality of research taking place [14, 15].

Development of a core outcome set for use in gastroschisis would standardise outcome reporting, reduce reporting bias, improve patient relevance and facilitate meta-analysis. Addressing these points would significantly improve the quality of research taking place and, when combined with improved collaboration between research institutions, allow for the development of evidence-based management guidelines.

## Methods/design

### Ethics and registration

The Health Research Authority deemed the project to be *Service Evaluation/Service Development* and therefore review by an NHS Research Ethics Committee is not necessary. Information on the nature of the study is provided to participants prior to registration and again prior to completion of the first round of the survey. Potential participants are given contact details for staff within the National Perinatal Epidemiology Unit from whom further information can be obtained or with whom they can discuss the study further. Detailed information is also available on the study website (www.npeu.ox.ac.uk/nets). Consent to participate in the study is implied by completion of the registration questionnaire and data-collection questionnaires. Participants can withdraw from the study at any time either by contacting the study team or by simply not completing a data-collection questionnaire. The study has been registered with the COMET initiative (www.comet-initiative.org).

### Scope of the core outcome set

The developed core outcome set is intended to be used to assess the overall success of treatment of an infant born with gastroschisis. This will involve outcome measures identified as important from birth into adulthood. The core outcome set is not intended to be applicable to antenatal interventions or interventions related to the mode or timing of delivery.

### Key objectives

1. Determine which outcomes are currently reported in studies comparing surgical treatments for gastroschisis and assess the quality of reporting
2. Prioritise outcomes from patient/parent, paediatric surgical and non-surgical clinician perspectives
3. Achieve consensus between key stakeholders on a core outcome set for assessing how successful the overall treatment of an infant with gastroschisis has been
4. Compare and contrast outcomes prioritised by patients/parents, surgeons and non-surgical clinicians

### Wider aims

1. A wider aim is to develop methodology for countering difficulties likely to be seen in the development of core outcome sets in all paediatric surgical conditions, including the following:
  - Recruitment of parents and patients
  - Incorporation of opinions from clinicians whose priorities vary dependent on their specialty or on the age at which they encounter the child.

### Design

Four key stages occur in the development of the core outcome set:

1. Systematic review to identify currently reported outcomes
2. Development of a panel of experts
3. Three-phase online Delphi process
4. Consensus meeting

### Systematic review

Two systematic reviews comparing methods of treatment for gastroschisis have been conducted. The first was a systematic review of broad scope, including both observational and experimental study designs (Allin BSR, Irvine A, Patni N, Knight M: Variability of outcome reporting in Hirschsprung’s Disease and gastroschisis: a systematic review, unpublished), whilst the second focussed on randomised controlled trials and systematic reviews of randomised controlled trials [16]. Outcome measures in these reviews will be used as the starting point for the development of the core outcome set. The developed core outcome set is intended to be relevant to post-natal interventions only. Therefore, any outcome measures identified from the systematic reviews that are deemed relevant only to antenatal interventions or interventions relating to mode and timing of delivery will not be carried forward to phase 1 of the Delphi process.

### Panel assembly – expert identification and recruitment

Panel assembly will be based on methods developed by Okoli et al. [17]. A knowledge resource nomination worksheet will be developed by members of the study management group. A knowledge resource nomination worksheet is used to ensure that experts are recruited across an adequate breadth of experience of gastroschisis. Initially, areas from which experts must be recruited will be identified under the headings of ‘Disciplines, Organisations and Literature’. Members of the study management group will populate each of these headings with categories of experts from which participants must be recruited. Examples of categories include ‘paediatric surgeons’*,* ‘parents’*, ‘*the Royal College of Paediatrics and Child Health’*,* and the ‘*Journal of Paediatric Surgery*’*.* Once the study management group has developed an exhaustive list of categories, an iterative process will be used to populate each category with names of potential experts. Initially, experts known to the study management group will be added to the knowledge resource nomination worksheet. Strategies to identify further experts in each category will then be developed. These strategies will differ from category to category, but some examples include those listed below.

#### Paediatric surgeons

Potential experts will include those identified as having an interest in managing infants with gastroschisis on a search of the British Association of Paediatric Surgeons register of practitioners. The clinical leads for each of the 27 paediatric surgical centres in the UK will be identified via the websites of those centres and will also be considered potential experts.

#### Neonatologists and paediatricians

Heads of department for all level 2 and level 3 neonatal units and their associated paediatric departments in the UK will be identified via the websites for these NHS trusts and will be considered potential experts.

#### Parents and patients

A parent advisory group has been established in the National Perinatal Epidemiology Unit. This group consists of parents of infants who have required early surgery, including those with gastroschisis, exomphalos, Hirschsprung’s Disease and anorectal malformations. Parents of infants with gastroschisis will be identified via the mailing list for this group. Additional parents/infants will be identified through use of the *Gastroschisis, Exomphalos, and Exstrophy Parent Support* mailing lists and Facebook groups. Members of the study management group, as well as the British Association of Paediatric Surgeons Congenital Anomalies Surveillance System steering committee, including BA, NH and SM, will be utilised to recruit infants and parents from their respective hospitals to give good representation from all regions in the UK. Parents/patients identified in each of these ways will be considered potential experts.

#### Journals

Editors of key paediatric and paediatric surgical journals will be considered potential experts. They will also be asked to identify any members of their editorial board who have expertise in gastroschisis.

Using similar strategies for each remaining category, the knowledge resource nomination worksheet will be populated with a master list of potential experts. Each person named on the knowledge resource nomination worksheet will be sent an information pack via email. In these information packs, we will explain that we have identified the recipient as someone with expertise in the management of infants with gastroschisis and that we are seeking to establish whether the recipient is be suitable for and interested in participating in development of a core outcome set. The information packs will also contain a plain language summary of the COMET initiative and a lay or scientific summary of the study as appropriate. A link will be provided to an online form for experts to express their interest in participation in the study and to provide more information on their involvement in management of infants with gastroschisis. Each expert will also be asked to provide the names and contact details of anyone else they believe would be suitable for inclusion in the study. At this stage, we are seeking to clarify the suitability of experts for participation in the study and to identify further potential experts. This process will be repeated for the names provided by the previously contacted experts. Expert recruitment will continue until a minimum of 50 experts, with at least two in each category, has been recruited.

Following confirmation of their eligibility to participate in the study, experts will be sent a link to a customised online database from which they can access phase 1 of the Delphi process.

### Scope of expert recruitment

An a priori decision has been made to limit recruitment of experts to those based in the UK.

### Panel assembly – facilitating consensus

Clinicians who treat gastroschisis in the neonatal period, such as paediatric surgeons, may have a different set of priorities than those who primarily manage affected children later in life, such as paediatricians. This may make it difficult to attain their consensus on a single set of outcome measures. It is essential that the core outcome set represent the views of patients/parents, neonatal clinicians and non-neonatal clinicians. To ensure that this occurs, experts will be separated into the following three panels:

- Neonatal panel – clinicians whose responsibility includes management in the neonatal period (but may also include management outside of the neonatal period). This group will include neonatologists and paediatric surgeons.
- Non-neonatal panel – researchers with expertise in gastroschisis management and clinicians responsible for management primarily outside of the neonatal period. This group will include specialist nurses and paediatricians.
- Lay panel – parents, and adults who were born with gastroschisis.

### Delphi process – phase 1 data collection

An online system will be used to conduct a three-phase Delphi process run in parallel for the ‘neonatal’ , ‘non-neonatal’ and ‘lay’ panels. An online system has been chosen in order to maintain anonymity, where experts do not know the names of other experts on their panel or other experts’ individual responses.

In phase 1a, participants will be presented in alphabetical order with the list of outcomes identified through the systematic reviews. The study management group will develop equivalent lay terms for each scientific outcome, and their understanding will be piloted with the National Perinatal Epidemiology Unit’s parent advisory group. These lay terms will be used instead of scientific terms in the questionnaires completed by the lay panel. If there is ambiguity over the outcome or domain, either in the scientific or lay questionnaire, ‘tips’ will be placed alongside them to clarify meaning.

Participants will be asked to give each outcome measure a score from 1–9 where 1, 2 and 3 are ‘not that important’; 4, 5 and 6 are ‘important’; and 7, 8 and 9 are ‘really important’.

The Grading of Recommendations Assessment, Development and Evaluation Working Group scale of measurement has been chosen for use in scoring outcome measures, based on recommendations from the COMET initiative [18].

Following completion of phase 1a, participants will be asked if there are any outcomes they consider important in determining whether treatment of their/their child’s/their patient’s gastroschisis has been successful, but which we have not yet identified. They will be able to list as many items as they consider necessary.

A planned period of 4 weeks will be scheduled for completion of data collection in phase 1 and for each subsequent phase. When experts have not returned their completed questionnaire within 2 weeks of the start of the phase, they will be contacted via email to remind them of the necessity to complete the phase. If they have not completed the questionnaire by the 3-week deadline, they will be contacted again via email to ascertain if they are having difficulties in completing the questionnaire or if they have decided they no longer want to participate in the study. Participants who have not completed the questionnaire within 4 weeks of the phase starting will be deemed not to have completed that phase.

### Delphi process – phase 1 analysis

The number of experts invited to participate, registering to participate, and completing phase 1 of the Delphi process from each category identified in the knowledge resource nomination worksheet will be recorded.

Two reviewers will independently assess outcomes reported in phase 1b in order to determine if they represent de novo outcomes not already listed in phase 1a. De novo outcomes listed by at least one expert will be taken forward to phase 2 of the Delphi process.

Outcomes will be analysed separately for each panel with descriptive statistics, including medians and inter-quartile ranges, being calculated. All outcomes will be carried forward to phase 2.

### Phase 2 data collection

Experts completing phase 1 will be invited to participate in phase 2 and asked to re-score each outcome based on the following:

- The phase 1 score they assigned it
- Descriptive statistics from their panel

Descriptive statistics will be represented numerically and graphically.

### Phase 2 analysis

Descriptive statistics will again be calculated. Bias from loss of experts between rounds will be assessed by determining if there is any difference in median round 1 scores for each outcome measure between experts who have completed both phases and experts who only completed phase 1. All outcomes will be carried forward to phase 3.

### Phase 3 data collection

Experts completing phase 2 will be invited to participate in phase 3 and will be asked to re-score each outcome based on the following:

- The phase 2 score they assigned it
- Round 2 descriptive statistics from all three panels

### Phase 3 analysis

Analysis will be conducted as per phase 2.

### Generation of core outcome set – consensus meeting

Experts who have completed all three phases of the Delphi process will be invited to the consensus meeting purposively with an even spread across panels and disciplines until 40 experts have confirmed their attendance. The intention of the consensus meeting is to ratify the established core outcome set, discuss outcomes where no consensus could be obtained and determine the most appropriate methods and timing for assessing the identified core outcomes. The consensus group will also discuss how identified core outcomes relate to the core areas within the OMERACT filter 2.0.

Following discussion of outcomes, each outcome will be re-scored anonymously and electronically by the meeting participants using the same scoring system as for the Delphi process. Participants will be asked to re-score outcomes based on their own scores, the results of the Delphi process and the discussions at the meeting. Outcomes reaching ‘consensus in’ as defined below following re-scoring at the consensus meeting will be included in the core outcome set. All others will be excluded.

Outcomes will be discussed at the consensus meeting in different groups according to the number of panels in which they have reached ‘consensus in’ at the end of the third phase of the Delphi process. ‘Consensus in’ will be defined as ≥ 70 % of participants rating the outcome 7–9, and < 15 % rating it as 1–3. Outcomes will additionally be classified as ‘consensus out’ if > 70 % participants rated it 1–3 and < 15 % rated it 7–9. All other outcomes will be considered as not achieving consensus in either direction. Only ‘consensus in’ outcomes will form part of the core outcome set.

Compliance with SPIRIT recommendations can be seen in the SPIRIT checklist (Additional file 1), and the SPIRIT Table (Table 1).

**Table 1:** SPIRIT Table

	Study period						
	Enrolment	Allocation to Panel 1, 2 or 3 According to Specialty	Post-allocation				Close-out
Timepoint	*December 2015-February 2016*	February 2016	*Round 1 (Feb–March 2016)*	*Round 2 April–May 2016*	*Round 3 May–June 2016*	*Consensus meeting 221 June 2016*	*July 2016*
Enrolment:	X						
Eligibility screen	X						
Implied consent through registration	X						
*(List other procedures)*							
Allocation		X					
Assessments:							
*(Outcome scores)*			X	X	X	X	

### Sub-group analysis

Paediatric surgeons are broadly responsible for determining which initial treatment strategy should be undertaken for infants with gastroschisis. They are also responsible for implementing this intervention. We are interested to see whether their views on outcomes of importance are different from those of clinicians whose primary role is in the treatment of the complications of gastroschisis or in monitoring of longer-term outcomes for infants with gastroschisis. In order to do this, we will perform a sub-group analysis comparing phase 3 scores for paediatric surgeons with phase 3 scores for non-surgical clinicians.

Following completion of the consensus meeting, a consensus document will be drafted and put forward to participants for approval. This document will be presented at appropriate international meetings and published in peer-reviewed journals.

### Data management

All data will be directly entered into a customised database by participants. Data will be stored securely on servers within the National Perinatal Epidemiology Unit and will be managed as per standard operating protocols. Only appropriate members of the study team will have access to the data. Data analysis will be conducted by BA and MK.

## Discussion

No core outcome set currently exists for use in determining how successful the overall treatment of a child’s gastroschisis has been. Development of one will standardise outcome measure reporting, thereby increasing patient relevance of research, reducing reporting bias, and increasing ease of data synthesis. Involvement of multiple key stakeholder groups in the development of the core outcome set will help ensure its validity and generalisability. Through implementation of the core outcome set, key clinical questions will be more readily answerable. Ultimately, this will aid identification of evidence-based treatments, improve outcomes for infants with gastroschisis and improve the quality of counseling of their parents.

### Study status

Participant recruitment has started.

## Abbreviations

COMET, core outcome measures in effectiveness trials; NHS, National Health Service; OMERACT, outcome measures in rheumatology

## Additional file

Additional file 1: Spirit Checklist. This file contains the completed SPIRIT checklist describing where key portions of text can be identified in the protocol. (DOC 121 kb)
